# Effects of Major Royal Jelly Proteins on the Immune Response and Gut Microbiota Composition in Cyclophosphamide-Treated Mice

**DOI:** 10.3390/nu15040974

**Published:** 2023-02-15

**Authors:** Wenqian Wang, Xiangxin Li, Dan Li, Fei Pan, Xiaoming Fang, Wenjun Peng, Wenli Tian

**Affiliations:** Institute of Apicultural Research, Chinese Academy of Agricultural Sciences, Beijing 100093, China

**Keywords:** major royal jelly proteins, non-labeled, quantitative proteomics technology, immune functions, 16S rDNA high-throughput sequencing technology, gut microbiota

## Abstract

Increasing evidence suggests that royal jelly (RJ) has exceptional biological properties, and that major royal jelly proteins (MRJPs) are the key active factors in RJ. The objective of this study was to compare the difference in the protein content between RJ and MRJPs using non-labeled, quantitative proteomics technology, and to investigate the adjustment features and mechanisms of MRJPs on murine immune functions and the composition of intestinal flora in cyclophosphamide-treated mice. Results showed that, during the process of extracting MRJPs, the ratio of the protein types in the main protein and other proteins decreased significantly, except for MRJP1 and MRJP7, which demonstrated that an enriching effect of MRJP1 and MRJP7 was present during the extraction process. Cyclophosphamide-induced mice were orally administered MRJPs. Results showed that the middle-dose group, which received 0.25 g/(kg·bw) of royal jelly main protein, demonstrated a clear impact on the development of the spleen and liver, the quantity of peripheral blood leukocytes, immunoglobulin content, immune factor level, and the proliferation ability of spleen lymphocytes. A 16S rRNA high-throughput sequencing technology analysis showed that MRJPs could improve the component and richness of intestinal flora and raise the immunity of mice. The above-mentioned results indicated that the application of MRJPs is very likely to have an advantage effect on murine immune functions.

## 1. Introduction

Recent research has increasingly concentrated on investigating the pharmacological properties of bee products, including honey, beeswax, bee venom, bee pollen, propolis, and royal jelly (RJ), which are composed of multiple substances and provide pharmaceutical and health-enhancing benefits. Among these bee products, RJ is a light yellow or creamy white, high-viscosity, fluid substance, which is produced by the hypopharyngeal and mandibular glands of juvenile worker bees [1]. It is the only bread of the queen bee for her whole life, and is given to the worker larvae in their inaugural three days; this difference decides the caste differentiation in a hive [2].

RJ is rich in nutrients and contains proteins (9–18%), free amino acids, carbohydrates (7–18%), phenols, fatty acids and lipids (3–8%), minerals (0.8–3%), vitamins, and other bioactive components [3]. As one of the most-preferred nutritional food products, RJ has been extensively used in food, medicine, cosmetics, and other fields [4,5] due to its antioxidant, antiproliferative, antimicrobial, and neuroprotective effects. Previous reports suggested that RJ protein was one of the most bioactive compounds in the RJ composition [4]. The major royal jelly proteins (MRJPs) possess important bioactive effects, including antioxidant, anti-bacterial, anti-tumor, anti-aging, cell-growth promoting, injury healing, neuroprotective, anti-inflammatory, and immunomodulatory agents, and account for 90% of the total proteins in RJ [6].

As a person’s life and work stress grows, poor habits and little regard for health results in diminished immune system function and corresponding physical symptoms [7]. The immune system is a prominent defense institution in the human body. It is comprised of immune organs, immune cells, and immune molecules. It prevents the invasion of pathogenic genes through the immune response and is essential in maintaining physiological balance [8,9]. Meanwhile, the intestine is the maximum digestive and absorptive organ, which also has vital defensive capabilities [10]. An unbroken intestinal flora shield is meaningful for maintaining the bodily physiologic shield and immune system [11]. In an otherwise healthy human body, the gut barrier plays a key role in the epithelial transport of nutrients and metabolites, as well as the prevention of damage from intraluminal substances [12]. Previous reports indicated that major royal jelly proteins could regulate immune function by stimulating macrophages and decreasing the number of inflammatory factors in a mouse body [13]. Previous studies showed that the major royal jelly proteins present a potent immune-regulating function. Monomeric MRJP1 and MRJP2 could stimulate mouse macrophages and promote the immune response of mice, both of which can stimulate macrophages to enhance the immune response, thus adjusting the system’s immune response level and achieving an immunostimulatory effect [14]. MRJP3 can inhibit anti-OVA IgE and IgG1 levels in the sera of intraperitoneally immunized mice, and soluble MRJP3 can reduce its antigenicity by heat treatment [15]. Cyclophosphamide (CTX) is a type of anticancer drugs often used to treat cancers and autoimmune diseases. However, it can cause serious immunosuppression and an imbalance of the gut microbiota. Therefore, MRJPs, which have an immune-protective benefit, are used to study the immune responses of mice induced by cyclophosphamide. The above results imply that MRJP1, MRJP2, and MRJP3 exhibit potent immunoregulatory effects. However, the modulatory action of MRJPs on immune functionality in mice via interactions with intestinal flora still requires further exploration.

Due to the significance of the gut microbiota in immunity-related diseases, it is of remarkable importance to investigate the useful impacts of MRJPs on the micro-ecological health of the gut. Based on these analyses, this study focuses on analyzing the difference in protein components between RJ and MRJPs. It also evaluates the immunoregulation influences of MRJPs on immunodeficient mice by regulating the influence of the immune organ, the release of immune factors, and the composition of intestinal microbiota in a cyclophosphamide-induced mouse model.

## 2. Materials and Methods

### 2.1. Chemicals and Samples

The royal jelly and MRJPs used in this study were obtained in Yan’an, Shanxi, China. Enzyme-linked immunosorbent assay (ELISA)-based cytokine kits (IgG, IgM, IL-4 and IL-6) and BCA kits were obtained from Meimian Industrial Co., Ltd. (Yancheng, China). A Power Soil DNA Isolation Kit was purchased from YMO BIO Laboratories, U.S.A. HPLC-grade acetonitrile was acquired from Fisher Chemicals (Fairlawn, NJ, USA). All other chemicals used in this study were of analytical grade.

### 2.2. Preparation of MRJPs

The MRJPs was extracted from the RJ as previously described, with some modification [16]. An amount of 5.0 g of fresh royal jelly was mixed with a phosphate buffer solution (0.5 M KH_2_PO_4_/K_2_HPO_4_ buffer, pH = 8.7) under stirring (4 °C, 300 rpm) for 1 h, followed by the addition of buffer to 50 mL. The mixed solution was centrifuged (4 °C, 12,000× *g* rpm) for 50 min. The supernatant was passed through a filter membrane with a 0.22 μm aperture; the filtrate was the water-soluble protein extract of the RJ. Eventually, the protein solution in the dialysis bag was removed and placed on a drying dish. It was then freeze-dried using a lyophilizer and preserved in a −20 °C refrigerator.

### 2.3. Proteomic Analysis

Extraction of proteins: An analysis of three biological replicates was processed in both ways with proteomics. The method of extracting protein from the RJ and MRJPs for proteomic analysis was described by previous reports, with some modifications [17]. Briefly, the RJ and MRJPs were each mixed with a Lysis buffer (1:1, *v*/*v*) and ultrasonicated for 2 min using an ultrasonic cell crusher at 4 °C. The above mixtures were allowed to centrifuge at 12,000× *g* rpm at 4 °C for 20 min, and the upper supernatant was mixed with three times the volume of cold acetone to precipitate the remaining proteins. This centrifugal operation was repeated three times. The supernatant was then removed, and the precipitate was collected. Then, 100 µL of 5 M urea was mixed into the precipitate, followed by ultrasonic treatment. Finally, 400 µL of NH_4_CO_3_ (40 mM) was mixed into the above solution.

Protein concentration determination: The method of determining the protein concentration referred to the previously reported study [18]. After the samples were diluted, the BCA kit was used to detect the protein concentration of the samples.

Proteolytic cleavage: An amount of 400 µg of RJ and MRJP protein extracts were mixed with 4 mL of DDT solution (0.1 M). After 1 h of reaction at chamber temperature, 2 mL of DDT (Dichlorodiphenyltrichloroethane) solution was added to the above mixture incubated in the dark for 1 h. Then, a trypsin enzyme with one-fifth of the protein mass was added for overnight cleavage. After 14 h, 1 µL of formic acid was mixed in to terminate the cleavage.

Peptide desalting and quantification: Desalting was performed using ZipTipC18 pipette tips, according to the standard procedure in the user guide [19]. 

The desiccated samples were solubilized in 0.1% formic acid water and centrifuged at 12,000× *g* rpm under chamber temperature for 20 min. After taking the supernatant, the peptide concentration was determined using a Nanodrop. It was then diluted to 0.25 µg/µL for mass spectrometry.

Mass spectrometry analysis:A peptide analysis was carried out using an HPLC system Easy-nLC1200 from Thermo Fisher Scientific (Waltham, MA, USA). This was coupled to a Thermo Orbitrap QE-HF mass spectrometer, according to previous reports [20]. 

Database searching and validation: Raw MS files were processed using MaxQuant, an in-house software suite developed in [21]. The search engine used was Andromeda, the search database used was Apis mellifera (from NCBI), and the contamination library used was the common contamination library sequence from MaxQuant software (version 1.6.1.0, Max-Planck-Institute of Biochemistry, Martinsried, Germany).

### 2.4. Animal Experiment

Eight-week-old Kunming mice, weighing 40–45 g, were acquired from the laboratory animal center of Tsinghua University (Beijing, China) and housed in cages with free access to food (Keao Xieli Feed Co., Ltd., Beijing, China) and water. The feeding conditions were an ambient temperature of 22 ± 2 °C a 12 h light/dark cycle. All animal experiments were carried out in accordance with related guidelines and regulations and with the authorization of the Institutional Ethical Committee of China (IACUC-20220124). Reporting guidelines were observed for all animal experiments [22]. 

Forty-eight mice (18 ± 2 g bw (body weight)) were acquired from Beijing Vital River Laboratory Animal Technology Co., Ltd (Beijing, China). The mice were reared suitably for the conditions (temperature, 23 ± 2 °C; relative humidity, 50–70%; 12 h/12 h light–dark cycle). After 3 days, these mice were divided randomly into six groups, including a normal group (NG) and a model group (MG) who received physiological saline for 30 days, and a casein group (MG + CG) who received casein (0.25 g/(kg bw)) for 30 days. The low-dose MRJP (MG + LM), medium-dose MRJP (MG + MM), and high-dose MRJP groups (MG + HM) were given MRJPs (0.125, 0.25, and 0.5 g/(kg·bw), respectively), administered intragastrically for 30 days. The MG, MG + CG, MG + LM, MG + MM, and MG + HM groups were intraperitoneally administered cyclophosphamide on days 28–30 (80 mg/(kg bw), while the NG group was administered saline in the same manner. The mice were sacrificed 24 h following the last gavage dose.

Body weight, sample collection, and organ index: At the end of 30 days, all mice were fasted overnight and anesthetized using ether. After weighing, they were then sacrificed according to animal ethics protocols. Blood and stool samples were gathered for biochemical analysis. The thymus and spleen tissue were separated and carefully washed with ultrapure water to remove RNase. The excess water was sucked out using filter paper, then weighed and collected. Calculations of thymus and spleen indexes were based on the following equation [23]:

Organ index (mg/g) = organ weight (mg)/body weight (g)

Histochemical examination of liver and spleen: A histochemical examination of the liver and spleen were carried out according to a previous study [23]. Spleens and livers from all groups were immobilized with 10% formalin, paraffin-embedded slices were colored with hematoxylin and eosin, and histological variations were recorded using light microscopy (Olympus Corporation, Tokyo, Japan).

White blood cell count: The number of white blood cells was measured using a Coulter LH 755 hematology analyzer (Beckman Coulter, Miami, Florida, USA), according to a previous study [24]. 

Immunoglobulin and cytokine assays: After euthanasia, blood samples were gathered from the orbits and centrifuged for 20 min at 1200 r/min at 4 °C to obtain the sera. IL-4, IL-6, IgM, and IgG concentrations were measured using ELISA kits, following the manufacturer’s instructions. (Varioskan Flash, Thermo Fisher Biochemical Products Co., Ltd., Waltham, MA, USA) [25].

Proliferation experiment of mouse spleen lymphocytes: According to the Taipan Blue assay, the cell viability was greater than 95%, and the cell density was set at 5 × 10^6^ cells/mL. An amount of 90 μL of mouse spleen lymphocyte suspension was inoculated into a 96-well cell-culture plate with or without 10 μL of ConA (5 μg/mL) or LPS (5 μg/mL). These were then incubated at 37 °C with 5% CO_2_ for 72 h. CCK-8 (5 μg/mL) was placed in each well, and the incubation was continued for 2 h. The optical density (OD) was then measured at 490 nm using a microplate reader (Thermo Fisher Scientific, Waltham, MA, USA) [23].

SI = OD_sample_/OD_blank_ × 100%

The blank group comprised mice lymphocytes fed with saline, and the proliferation ability of the lymphocytes was evaluated by stimulation index (SI).

### 2.5. Intestinal Flora Analysis

Feces samples were collected for intestinal flora analysis.

DNA isolation and ITS PCR: Total fungal DNA was isolated from fecal samples with a Power Soil DNA Isolation Kit, depending on the manufacturer’s recommendations [26].

Sequence processing: A high-throughput sequencing analysis of fungi was conducted using the Illumina Hiseq 2500 platform. The raw paired-end reads (PE reads) for an individual sample were combined using FLASH v1.2.7, and the high-quality, clean labels were filtered by the use of raw tags splicing using a Trimmomatic, v0.33. Valid tags were obtained by stripping chimeric sequences using UCHIME v4.2. All valid tags were classified into an operational taxonomic unit (OTUS) based on the QIME (version 1.8.0), with a 97% sequence similarity.

Species distribution and correlation analysis: The components of the individual zones at the phylum and genus levels were statistically examined. Differences in microbial group abundance between the groups were detected using Metastats software (Version 2.0, Center for Bioinformatics and Computational Biology, Newark, NJ, USA), followed by *t*-tests for comparative species richness among the six groups. The *p*-value was derived from the *t*-test, and the *q*-value was the modified value of the *p*-value. Six groups of samples were filtered for differences in species composition using q values. An associative analysis (for both positive and negative associations) was conducted using the Spearman association analysis. Association networks were plotted using a Python program, based on association coefficients >0.1 and *p*-values < 0.05.

### 2.6. Statistical Analyses

Each experiment was performed a minimum of three times. Data are shown as the average ± standard deviation (SD). SPSS version 22 (IBM, Armonk, NY, USA) and GraphPad Prism Version 7 (Graph Pad Software Inc., Boston, MA, USA) were applied to analyze and handle data. All analyses were conducted using a one-way ANOVA and Tukey’s test, and results at *p* < 0.05 were regarded as remarkable.

## 3. Results and Discussion

### 3.1. Quality Evaluation of RJ Protein and MRJPs 

**Figure 1:**
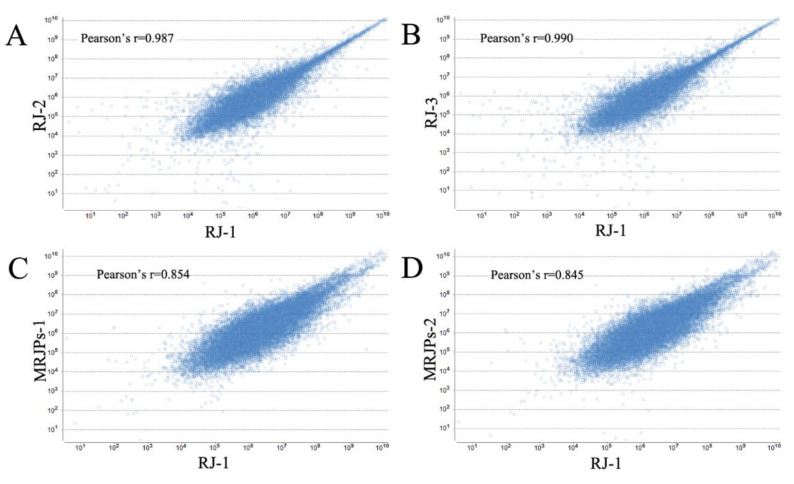

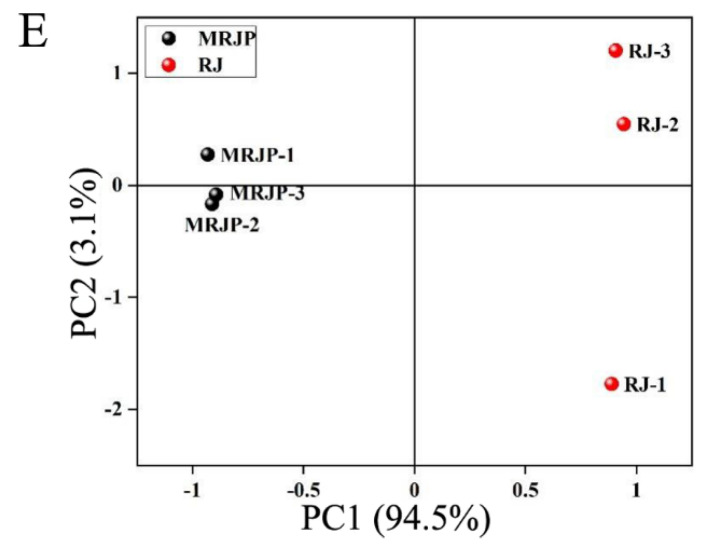
Pearson correlation coefficient and principal component analysis between the royal jelly and MRJPs with three times repeated data. (**A**) Pearson correlation coefficient between RJ-1 and RJ-2; (**B**) Pearson correlation coefficient between RJ-1 and RJ-3; (**C**) Pearson correlation coefficient between RJ-1 and MRJPs-1; (**D**) Pearson correlation coefficient between RJ-1 and MRJPs-2; and (**E**) principal component analysis between RJ and MRJP with three times repeated data.

The reliability and rationality of the samples, including the different repetitions of the same sample and the correlation between different samples, must be evaluated. Pearson’s correlation coefficients were employed to describe the correlation of the two variants. The reliability and accuracy of the obtained proteome data were evaluated using PEAKS software (version 8.5, Bioinformatics Solutions Inc., Waterloo, ON, Canada). As is shown in Figure 1A,B, for each biological duplication of the RJ protein, the Pearson correlation coefficient between them is more than 0.97, indicating that the RJ protein samples demonstrated good repeatability. As is illustrated in Figure 1C,D, the correlation coefficient between the RJ and MRJP samples is more than 0.82, implying that there is more difference between the RJ and MRJP samples. The above results suggest that the instrument was stable and the data were reliable. 

A PCA (Principal component analysis), an unmonitored visual method, was applied to decrease the dimensionality of the primary data and to assess the discrepancies among the samples [27]. As is shown in Figure 1E, the PCA analysis indicated the same results as the above description. Two principal components were extracted from six samples. There was a marked difference between the royal jelly samples and the MRJP samples, while the close distance of the same samples indicated that the samples were replicated well.

### 3.2. Protein Identification and Quantification

The proteomic differences between the royal jelly and MRJPs were compared by high-performance liquid chromatography/mass spectrometry (HPLC/MS) in tandem, and the changes in the composition and content of the MRJPs were further clarified during the extraction process.

**Figure 2:**
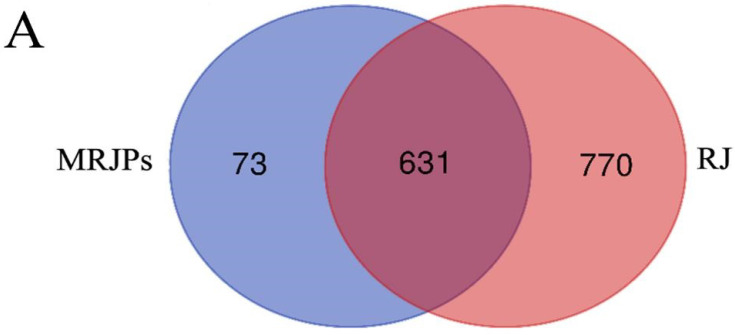

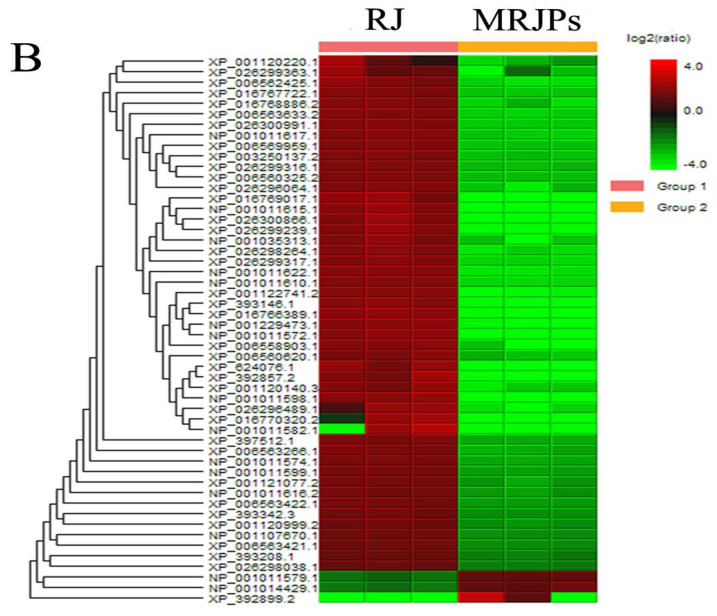
Protein identification and quantification analysis. (**A**) Venn diagram of shared and unique peptides of royal jelly and MRJPs; (**B**) protein cluster analysis of differences in content between royal jelly and MRJPs (fold change >1.5, *p* value < 0.05).

As is shown in Figure 2A, for protein identification, 1401 different peptides were obtained from royal jelly samples, while 704 different peptides were obtained from the MRJP samples. Among these peptides, 631 peptides were identified by both, 770 peptides were unique to royal jelly, and 73 peptides were unique to MRJPs.

As is shown in Figure 2B, a quantitative comparison of the RJ and MRJPs showed that a total of 52 proteins differed in content, with 49 of the MRJPs decreasing in content compared to the royal jelly, accounting for 94% of the total difference, and 3 proteins increasing in content, accounting for 6% of the total difference, including major royal jelly protein 1 precursor (*Apis mellifera*), major royal jelly protein 7 precursor (*Apis mellifera*), and heat shock protein 60A (*Apis mellifera*).

There are ten members of the MRJP family thus far (MRJPs1–9 and MRJP-ψ). This was verified at the protein or cDNA level. Combined with Table S1, it was concluded that the MRJP1 and MRJP7 in the main protein are enriched after purification.

### 3.3. Body Weight and Organ Index Analysis of Mice

**Figure 3:**
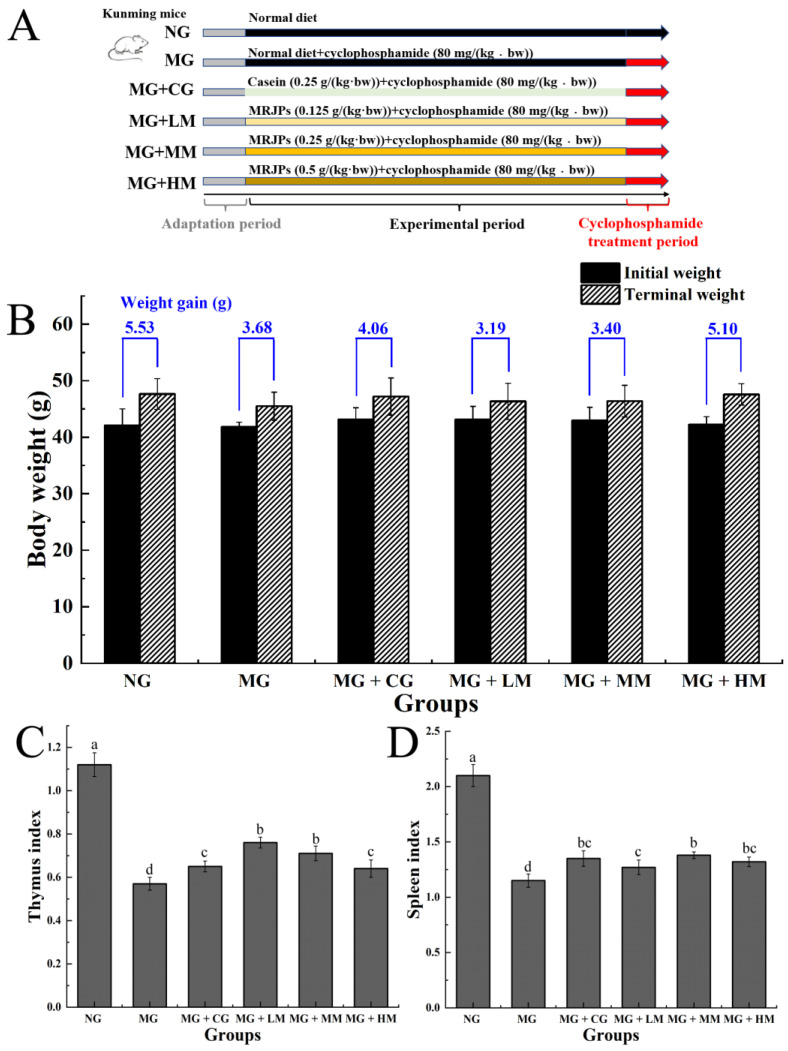
Experiment design and influences of MRJPs on the body weight and organ index of cyclophosphamide-induced, immunodeficient mice. (**A**) Animal experimental design; (**B**) influences of MRJPs on body weight of cyclophosphamide-induced, immunodeficient mice; (**C**) influences of MRJPs on thymus index of cyclophosphamide-induced, immunodeficient mice; and (**D**) effects of MRJPs on spleen index of cyclophosphamide-induced, immunodeficient mice. The different letters above each bar indicate statistically significant differences (*p* < 0.05).

The experimental design is shown in Figure 3A. The weights of the MG, MG + CG, MG + LM, MG + MM, and MG + HM groups were no different from the weights of the NG group (*p* > 0.05) (Figure 3B). After the end of gavage administration, the body weight increases of the MG, MG + CG, MG + LM, MG + MM, and MG + HM groups were less than that of the NG group (*p* > 0.05). When compared with the MG groups, the weight increase of each dose group of MRJPs was not notable (*p* > 0.05). The outcomes indicated that the immunocompromised mouse model was successfully constructed; this also indicated that the selected doses of cyclophosphamide were highly toxic to mice. Similar results were reported in the previous literature [28]. As is shown as Figure 3C,D, the thymus and spleen indices of the MC group were dramatically lower (*p* < 0.05) than those of the NC group. Nevertheless, the treatment with MRJPs enhanced the thymus exponent of the immunodeficient, cyclophosphamide-induced mice (*p* < 0.05). The above results also indicated that MRJPs contributed to an improvement in the immunity of immunodeficient, cyclophosphamide-induced mice.

### 3.4. Histological Observations of Spleen and Liver

**Figure 4:**
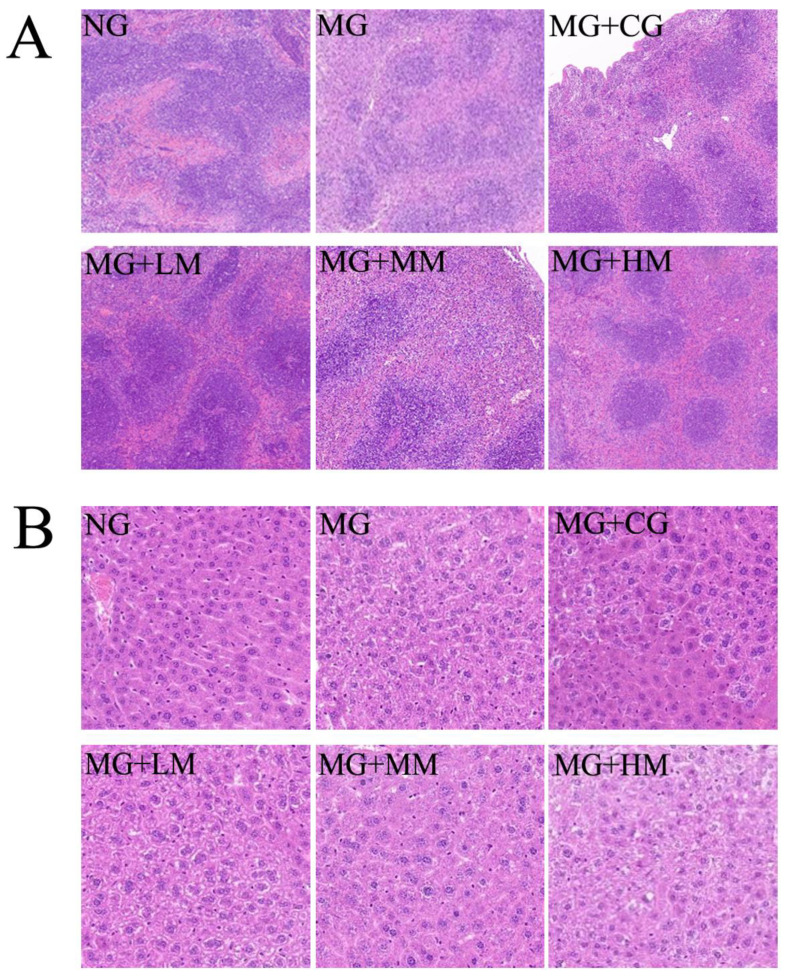
Effect of MRJPs on H&E staining of the spleen and liver. (**A**) Histological observations of spleen and (**B**) histological observations of liver.

The histopathological evaluation of the spleen is illustrated in Figure 4A. The spleen is among the most vital immune organs in the body, and the structure of immune organs mainly indicates the level of immune response in the body. After H&E staining, the spleen tegument of the mice in the NG was thicker and stretched into the parenchyma to establish trabeculae. The splenic parenchyma consisted of a well-defined red and white marrow, with the white marrow being more densely populated with lymphocytes. By comparison, in the H&E-stained sections of the spleen tissues of mice in the MG, the mouse spleens were disorganized, a large number of splenic nodules were destroyed, the red and white medullae were poorly defined, the white medullae were scattered, and the germinal center was scattered. However, after processing with MRJPs, the spleen tegument of the mice showed a tendency to be intact, the area of white marrow increased clearly, the germinal center converged, and the structure was intact and distinct.

The histopathological analysis of the liver is shown in Figure 4B. Previous research demonstrated that the content of immune cells in the liver is greater, and that the health of the spleen is related to the health of the liver. Additionally, because the liver is connected to the spleen through the hepatic portal vein, spleen injury could inevitably cause liver injury. No pathological abnormalities were found in the liver sections of mice in the NG. The liver cells were radially arranged around the central vein and plump and neatly arranged, and hepatic sinuses were clearly visible. In addition, clear nuclei could be seen with relatively complete boundaries, and the size of the nuclei was basically uniform and evenly distributed in the liver cells. By comparison, in the H&E-stained sections of the liver tissues of mice in the MG group, the partial liver nuclei were irregularly shaped and densely distributed surrounding the central vein. The liver cell volume was significantly enlarged and loosely arranged, with more vacuoles. A significant amount of cell necrosis could be observed in the liver cells of the MG + HM group. After treatment with MRJPs, the vacuoles of liver slices were strikingly reduced, and the structure of liver cells was greatly improved.

### 3.5. Determination of White Blood Cells in Blood, Immunoglobulin and Cytokine in Serum, and the Proliferation of Spleen Lymphocytes

**Figure 5:**
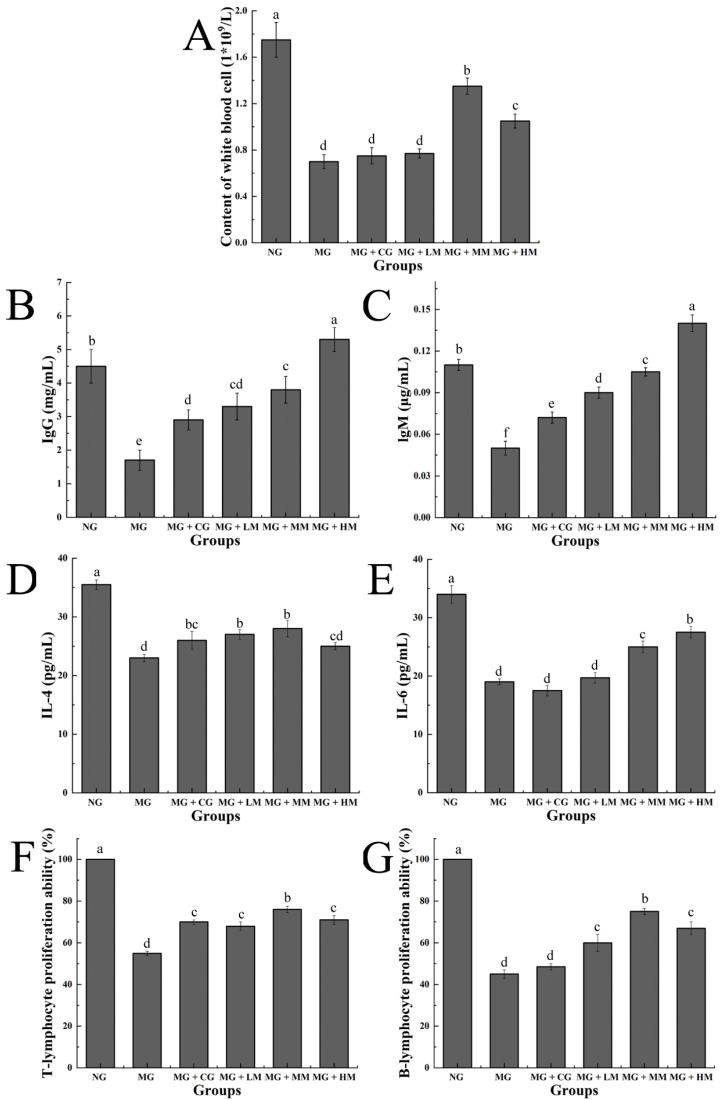
Effects of MRJPs on the immune indices of mice. (**A**) Content of leukocyte; (**B**) concentration of IgG; (**C**) concentration of IgM; (**D**) concentration of IL-4; (**E**) concentration of IL-6; (**F**) T-lymphocyte proliferation ability; and (**G**) B-lymphocyte proliferation ability. The different letters above each bar indicate statistically significant differences (*p* < 0.05).

Leukocytes play invaluable functions in fighting inflammatory disorders, bacterial infections, and cancers due to their specific phagocytic function. Therefore, an increase in leukocyte levels can improve the level of immune response and diminish the probability of disease [29]. As is illustrated in Figure 5A, when compared with the NG group, the levels of white blood cells in the MG group were markedly reduced (*p* < 0.05), which suggested that the cyclophosphamide substantially disturbed the health of the mice. When compared with the MG group, the white blood cell content of the MG group showed no difference. After treatment with the MRJPs, the leukocyte content of the MG + MM and MG + HM groups significantly increased (*p* < 0.05), indicating that MRJPs could enhance the immunity of mice by alleviating the decrease in leukocyte levels that was obviously induced by the cyclophosphamide.

IgG and IgM levels are regarded as biomarkers of the status of the humeral immune response, generating antibodies in the humoral immune response [30]. As is illustrated in Figure 5B,C, when compared to the NG, the serum IgG and IgM levels in the MG group were dramatically reduced (*p* < 0.05). Nevertheless, these levels exhibited increasing levels in the treatment group to varying degrees (*p* < 0.05), and exhibited a dose-dependent mode. These findings indicated that MRJPs augment the humoral immunity of mice by increasing the levels of IgG and IgM obviously induced by cyclophosphamide.

Cytokines are a class of biologically active substances produced by immune cells for the regulation and maintenance of immune homeostasis in the human body [31]. As is shown in Figure 5D,E, the levels of IL-4 and IL-6 in the MG group were lower than those in the NG group (*p* < 0.05). When compared to the MG group, MRJPs markedly increased the levels of IL-4 and IL-6 in the mice sera from the treatment groups (MG + CG, MG + LM, MG + MM, and MG + HM). These results indicated that MRJPs can improve the immune system response by regulating immune cells to secrete cytokines to further influence the metabolic pathway.

Lymphocyte proliferation is a critical index for the analysis of cellular immunity. It is a common method for detecting the immune-enhancing activity of drugs [32]. T/B lymphocytes are associated with the immune level of the body, which reflects the response levels of humoral and cellular immunity. As is illustrated in Figure 5F, compared with the NG, the proliferation of T lymphocytes in the spleen of the MG group was markedly inhibited (*p* < 0.05), demonstrating that the specific immune feature of the mice was affected and the modeling was successful. Compared with the MG group, the proliferation of T lymphocytes in the spleen of the mice treated with various doses of the main protein was significantly enhanced (*p* < 0.05). As is shown in Figure 5G, the findings indicated that the proliferation ability of B lymphocytes in the spleen of mice with the middle and high dose intake was remarkably enhanced (*p* < 0.05), but the effect of the low-dose treatment was not evident. Therefore, the MRJPs significantly enhance the proliferation of splenic lymphocytes in immunosuppressed mice.

### 3.6. Analysis of the Structure of Gut Microbiota in Mice

**Figure 6:**
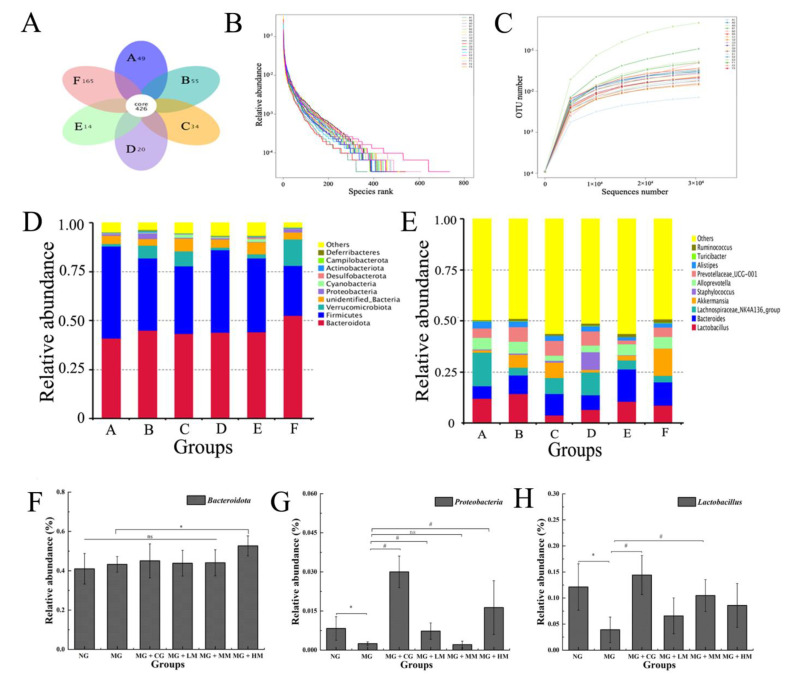
Impact of MRJP administration on intestinal microbiota in cyclophosphamide-induced mice. (**A**) The effect of MRJPs on the number of intestinal microbes OUT—A. NG; B. MG + CG; C. MG; D. MG + LM; E. MG + MM; and F. MG + HM; (**B**) rarefaction curve; (**C**) OTU rank curve of fecal DNA samples; (**D**) relative abundance of microbes in phylum; (**E**) relative abundance of microbes in genus; (**F**) relative abundance of *Bacteroidota*; and (**G**) relative abundance of *Proteobacteria*. (**H**) relative abundance of *Lactobacillus*.The different letters above each bar indicate statistically significant differences (*p* < 0.05).

In order to investigate the influence of MRJPs on the intestinal microbiota, fecal samples from each group of mice were analyzed using 16S rDNA sequencing [33]. An analysis of the common and distinct number of OTUs between different samples (groups). based on the clustering results of operational classification units (OTUs), was performed [34]. As is illustrated in Figure 6A, there were 426 OTUs in the six groups. The number of OTUs in the NG, MG, MG+CG, MG + LM, MG + MM, and MG + HM groups was 49, 34, 55, 20, 14, and 165, respectively. The above results indicated that, after cyclophosphamide treatment, the conformation of the intestinal microbial flora in mice decreases significantly, and high-dose treatments using MRJP obviously improves the intestinal flora structure of immunodeficiency mice.

The species diversity can be represented by the OTU rank curve, which covers the two dimensions of species richness and evenness [35]. The length of the horizontal axis of the curve in the figure indicates the richness of species in the sequencing samples. The length of the vertical axis of the curve indicates the species evenness in the sequenced samples. As is shown in Figure 6B, the OTU rank curve of the MRJP treatment groups was above the curves of the NG and MG groups, and the horizontal axis of the curve of the treatment groups was longer, indicating that the intestinal flora of these mice were enriched in the MRJP treatment groups, and was more than that of the mice in the model group and the blank group.

The dilution curve is another manifestation of sample diversity [36]. The dilution curve can be used to evaluate the sequencing depth of the sample. As is shown in Figure 6C, the species abundance and uniformity indicated in the rank abundance curve, which represented the sample sequence, was adequate for data analysis.

According to the species annotation, the top ten species with the highest abundance at the taxonomic level were selected, and a histogram was formed of the relative abundance of species. It is possible to compare the changes in the relative richness of the species in the various samples. As is shown in Figure 6D, according to the relative species multiplicity analysis, the top ten phyla were *Bacteroidota*, *Frimicutes*, *Verrucomicrobia*, *Unidentified-Bacteria*, *Proteobacteria*, *Cyanobacteria*, *Desulfobacterota*, *Actinobacteria*, *Campilobacterota*, and *Deferribacteres*. *Bacteroidetes* and *Frimicutes* were the two dominant species in the intestinal tract. *Bacteroidetes* is the largest, Gram-negative phylum in the human intestine, and it is regarded as having a key role in the host colonic environment to maintain healthy and intricate homeostasis. Gut ecological dysbiosis may change the host’s immunity and metabolism [37,38]. Compared with the MG group, the proportion of *Bacteroidetes* in the MG + HM group was remarkably raised (*p* < 0.05), suggesting that the high dose of MRJP treatment augmented the immunity of the mice by regulating the abundance of *Bacteroidetes* in the gut (Figure 6F).

As is shown in Figure 6E, at the genus level, the top ten genera exhibiting a variation in richness among the six groups were *Lactobacillus*, *Bacteroides*, *Lachnospiraceae*, *Akkermansia*, *staphylococcus*, *Alloprevotella*, *Prevotellaceae*, *Alistipes*, *turicibacter*, and *Ruminococcus*. Changes were found in the relative richness of *Lactobacillus* in the mouse guts. Compared with the NG group, the number of *Lactobacillus* reduced clearly in the MG group (*p* < 0.05). After the intragastric administration of MRJPs, the bacterial richness in mice shifted towards normal levels, particularly in the MG + MM group, for which the richness approached that of the drug group.

## 4. Conclusions

In conclusion, the non-labeled, quantitative proteomics results showed that, after the protein extraction process, MRJP1 and MRJP7 were enriched in MRJPs. The establishment of an immunosuppressive model in mice was used to evaluate the immune regulation action of MRJPs. The findings indicated that MRJPs could regulate immune function in cyclophosphamide-induced mice by upregulating the content of white blood cells, the concentration of IL-4 and IL-6, and the T/B-lymphocyte proliferation ability. These findings indicated that MRJPs could augment the cyclophosphamide-induced immune feature of immunosuppressed mice. Furthermore, this research is the primary study to explore the mechanism of the immune-potentiating role of MRJPs via regulation of gut microbiota. This research also suggested that MRJPs play a main role through the modulation of immune-associated intestinal bacteria. These findings will contribute to the exploration of the mechanisms by which MRJPs present immune-regulation roles, as well as supply a study basis for the further development of new functions and products.

## Data Availability

The data supporting the conclusions of this article obtained from the corresponding author upon request.

## Supplementary Materials

The following supporting information can be downloaded at: https://www.mdpi.com/article/10.3390/nu15040974/s1, Table S1: Royal jelly and main protein content difference protein list.
