# Supplementary material for: Effects of Major Royal Jelly Proteins on the Immune Response and Gut Microbiota Composition in Cyclophosphamide-Treated Mice
Source: Nutrients. 2023 Feb 15;15(4):974. doi: 10.3390/nu15040974 (PMC9967945; doi:10.3390/nu15040974)
Supplement: Supplementary file 1 [file nutrients-15-00974-s001.zip › nutrients-2135987-supplementary.pdf]

Table S1. Royal jelly and main protein content difference protein list.

Protein ID	Accession	RJ Area	MRJP Area	Group Profile (Ratio)	Avg. Mass	Description
1163012	XP_026299316.1	4.57E+08	6.65E+07	1.00:0.15	41096	major royal jelly protein 5 isoform X1 [Apis mellifera]
1163011	NP_001011599.1	2.40E+09	5.89E+08	1.00:0.25	70236	major royal jelly protein 5 precursor [Apis mellifera]
702679	XP_026299317.1	4.86E+08	4.10E+07	1.00:0.08	52829	major royal jelly protein 4 isoform X1 [Apis mellifera]
1163006	NP_001011610.1	1.23E+09	9.86E+07	1.00:0.08	52916	major royal jelly protein 4 precursor [Apis mellifera]
524105	NP_001011574.1	4.61E+08	9.78E+07	1.00:0.21	67938	glucose oxidase [Apis mellifera]
574391	XP_006560325.2	1.25E+08	1.76E+07	1.00:0.14	119831	uncharacterized protein LOC102655185 isoform X1 [Apis mellifera]
542304	NP_001107670.1	2.12E+08	6.70E+07	1.00:0.32	21348	apolipophorin-III-like protein precursor [Apis mellifera]
533144	NP_001011616.2	9.79E+08	2.60E+08	1.00:0.27	10717	defensin-1 preproprotein [Apis mellifera]
534545	XP_026298038.1	3.98E+08	1.65E+08	1.00:0.42	72229	uncharacterized protein LOC413627 [Apis mellifera]
546947	XP_397512.1	5.49E+07	1.08E+07	1.00:0.20	19434	uncharacterized protein LOC408608 [Apis mellifera]
1163015	XP_006563633.2	5.61E+07	5.37E+06	1.00:0.10	18361	uncharacterized protein LOC726323 isoform X2 [Apis mellifera]
515487	XP_006563266.1	6.56E+07	1.29E+07	1.00:0.20	24490	icarapin-like isoform X1 [Apis mellifera]
522467	XP_016767722.1	3.16E+07	3.70E+06	1.00:0.12	69262	glucose dehydrogenase [FAD quinone] isoform X2 [Apis mellifera]
812131	NP_001014429.1	8.45E+08	1.85E+09	1.00:2.19	50541	major royal jelly protein 7 precursor [Apis mellifera]
870545	XP_006569959.1	1.48E+07	1.85E+06	1.00:0.13	42665	venom acid phosphatase Acph-1 [Apis mellifera]
1204300	XP_016766389.1	6.37E+06	7.38E+04	1.00:0.01	35907	uncharacterized protein LOC408666 [Apis mellifera]
909063	NP_001011622.1	2.02E+07	1.29E+06	1.00:0.06	49786	major royal jelly protein 6 precursor [Apis mellifera]
515154	NP_001011579.1	1.07E+10	2.09E+10	1.00:1.96	48886	major royal jelly protein 1 precursor [Apis mellifera]
1224225	NP_001011582.1	8.84E+06	0	1.00:0	7946	apisimin precursor [Apis mellifera]
1163570	XP_006563421.1	8.30E+07	2.69E+07	1.00:0.32	8083	chymotrypsin inhibitor-like [Apis mellifera]
519970	XP_001120140.3	5.67E+06	5.25E+05	1.00:0.09	16794	NPC intracellular cholesterol transporter 2 homolog a [Apis mellifera]
831655	XP_001122741.2	4.03E+06	1.11E+05	1.00:0.03	30191	uncharacterized protein LOC727028 [Apis mellifera]
514221	XP_026296064.1	4.58E+06	5.36E+05	1.00:0.12	116165	lysosomal alpha-mannosidase isoform X2 [Apis mellifera]
515422	XP_016768886.2	5.59E+06	6.01E+05	1.00:0.11	142368	xanthine dehydrogenase [Apis mellifera]
1177004	NP_001011598.1	5.13E+06	0	1.00:0	55947	alpha-amylase precursor [Apis mellifera]
513971	NP_001229473.1	4.46E+06	5.85E+04	1.00:0.01	22644	peptidyl-prolyl cis-trans isomerase B precursor [Apis mellifera]
571638	NP_001011572.1	3.06E+06	0	1.00:0	78657	transferrin 1 precursor [Apis mellifera]
924261	NP_001011615.1	2.91E+06	0	1.00:0	14492	hymenoptaecin preproprotein [Apis mellifera]
1163573	XP_003250137.2	8.53E+06	1.15E+06	1.00:0.13	26430	cell wall integrity and stress response component 1 [Apis mellifera]
524907	XP_006562425.1	2.04E+06	1.49E+05	1.00:0.07	50736	serine protease inhibitor 88Ea isoform X1 [Apis mellifera]
643994	XP_006558903.1	1.04E+07	4.60E+05	1.00:0.04	52947	carboxypeptidase Q [Apis mellifera]
513704	XP_026300991.1	8.15E+06	8.90E+05	1.00:0.11	25603	ferritin subunit [Apis mellifera]
1204319	XP_393146.1	8.78E+05	1.05E+04	1.00:0.01	22953	uncharacterized protein LOC409648 [Apis mellifera]
515318	XP_026298264.1	1.37E+06	1.04E+05	1.00:0.08	18920	superoxide dismutase [Cu-Zn] chloroplastic-like [Apis mellifera]
513455	XP_392857.2	4.94E+05	0	1.00:0	42222	lysosomal aspartic protease [Apis mellifera]
513357	XP_016769017.1	7.90E+06	2.02E+05	1.00:0.03	49330	chitinase-like protein EN03 isoform X1 [Apis mellifera]

924479	NP_001011617.1	7.27E+06	7.56E+05	1.00:0.10	5903	abaecin precursor [Apis mellifera]
513090	XP_392899.2	0	2.76E+05	00:01.0	60413	heat shock protein 60A [Apis mellifera]
790687	XP_393208.1	1.40E+07	5.68E+06	1.00:0.40	58571	putative glucosylceramidase 4 [Apis mellifera]
1163143	XP_001120999.2	1.08E+07	3.42E+06	1.00:0.32	10072	chymotrypsin inhibitor [Apis mellifera]
1204301	XP_016770320.2	4.18E+05	0	1.00:0	61799	esterase B1 [Apis mellifera]
791674	XP_026299239.1	7.38E+05	0	1.00:0	32185	uncharacterized protein LOC113219062 [Apis mellifera]
516882	XP_006560620.1	3.94E+06	5.35E+05	1.00:0.14	39483	venom serine protease Bi-VSP [Apis mellifera]
532567	XP_393342.3	3.49E+06	1.12E+06	1.00:0.32	82918	transcription factor SPT20 homolog [Apis mellifera]
681364	XP_026299363.1	2.18E+06	5.05E+05	1.00:0.23	99906	trichohyalin isoform X1 [Apis mellifera]
532894	XP_026296489.1	1.07E+06	6.56E+04	1.00:0.06	50392	alpha-N-acetylgalactosaminidase [Apis mellifera]
514025	NP_001035313.1	1.02E+06	9.28E+04	1.00:0.09	15201	odorant binding protein 14 precursor [Apis mellifera]
515492	XP_624076.1	6.57E+05	0	1.00:0	25186	ferritin heavy polypeptide-like 17 [Apis mellifera]
591629	XP_001120220.1	5.01E+06	9.56E+05	1.00:0.19	16123	NPC intracellular cholesterol transporter 2 [Apis mellifera]
1163571	XP_001121077.2	1.12E+07	2.88E+06	1.00:0.26	9315	chymotrypsin inhibitor [Apis mellifera]
516350	XP_006563422.1	1.18E+07	3.09E+06	1.00:0.26	8130	chymotrypsin inhibitor [Apis mellifera]
